# Descriptive Review of Online Information Resources for People With Stroke: Protocol for a Scoping Review

**DOI:** 10.2196/23174

**Published:** 2021-07-13

**Authors:** Gakeemah Inglis-Jassiem, Karen Grimmer, Thandi Conradie, Quinette Louw

**Affiliations:** 1 Division of Physiotherapy, Department of Health and Rehabilitation Sciences Faculty of Medicine and Health Sciences Stellenbosch University Cape Town South Africa

**Keywords:** stroke, online resources, content, readability, design

## Abstract

**Background:**

People with stroke and their caregivers experience numerous information needs; internet-based resources may offer cost-effective ways to improve access to information about this condition and its management, including the availability of resources and support. The quality of online health information is, therefore, an important consideration for both developers and consumers of these online resources.

**Objective:**

This study aims to map and evaluate the content, readability, understandability, design, and quality characteristics of freely available online information resources (ie, websites) that empower people with stroke and their caregivers with information and self-help strategies poststroke.

**Methods:**

This descriptive review will follow the five systematic and rigorous methodological steps that are recommended for scoping reviews, which include the following: (1) identifying the research question, (2) identifying relevant studies, (3) selecting the studies, (4) charting the data, and (5) collating, summarizing, and reporting the results. Data will then be synthesized and analyzed thematically.

**Results:**

As of February 2021, the scoping review is in the data extraction stage. Data will be synthesized, and the first results are expected to be submitted for publication in an open-access peer-reviewed journal in August 2021. In addition, we will develop an accessible summary of the results for stakeholder meetings. Ethical approval is not required for this review, as it will only include publicly available information.

**Conclusions:**

This study is novel and will evaluate the typology, content, and design-related criteria, including accessibility, aesthetics, navigability, interactivity, privacy, and data protection, of online information resources for stroke. The review will be limited to online resources published in English.

**International Registered Report Identifier (IRRID):**

DERR1-10.2196/23174

## Introduction

Online, internet, or web-based stroke resources could be beneficial for people with stroke and their caregivers, specifically for those with information and support needs living in the community who have limited access to stroke care and rehabilitation. Apart from the benefit of easy access, users or consumers of these online resources may still experience unmet needs if other quality criteria, such as accuracy and readability, are not in place [1,2].

Stroke is the second most common cause of death [3], accounts for 102 million disability-adjusted life years (DALYs) globally [4], and is ranked as the third most common cause of DALYs worldwide [5]. In Africa specifically, stroke is increasingly “becoming a public health problem...as it causes high rates of disability and mortality” [6]. Motor-sensory dysfunction that impairs functioning, such as activities of daily living, transfers, and mobility, are concerning sequalae of stroke. In addition, depression, social isolation, and not being able to return to work and remain socioeconomically active citizens are also of great concern for people with stroke. The high levels of disability and care needs of stroke survivors impose a significant burden on carers who are often family members, resulting in a disruption of family functioning. Meeting the need for effective and efficient health and rehabilitation services poststroke is imperative to optimize functioning poststroke and to reduce the burden of care [7]. A recent review found that stroke rehabilitation services in Africa are generally poor, with challenges including “fragmented services, lack of trained personnel, and infrastructure limitations,” which lead to incomplete social and community reintegration of people with stroke in these resource-constrained settings [6].

Other than rehabilitation interventions, stroke patients and caregivers, globally, experience numerous diverse information needs after the incident, many of which are not met [8]. These needs include knowledge about the clinical aspects of stroke, prevention, treatment, functional recovery, and support services. Commonly reported needs of caregivers are related to transfers, exercises, psychological changes, and nutritional issues. With these needs addressed, people with stroke and their caregivers are empowered with information and strategies, which reduces psychosocial distress and caregiver burden and enhances self-efficacious behavioral changes [8]. Those affected by stroke will benefit from information tailored to their situational needs [8], but it is recognized that the provision of information and support remains poorly addressed globally [9]. Within resource-constrained environments, many barriers exist to equitable health care and rehabilitation service delivery and access [10]. Online or internet-based resources may offer cost-effective ways to improve awareness of, and access to, existing stroke care services, as well as self-management support for users of these digital health interventions [11].

The internet, or the World Wide Web, is increasingly being used for health-related inquiries by the general public [1]. There is also a growing body of literature into the use of technology as an adjunct or replacement for face-to-face stroke rehabilitation, education, and self-management strategies [12,13]. Digital health in stroke may take the form of various technologies, including websites; computer software; health apps, which could be used on mobile devices, such as smartphones or tablets; and even gaming consoles. Overall, online information on health conditions and their management has become accessible and inexpensive, which attracts the general public to use it for self-diagnosis or management [1], sometimes well before seeking assistance from health care professionals. A few benefits for users of using digital health include patient empowerment and engaging patients in their own health care [14]. Being able to source information based on recognized health needs not only empowers patients or users with information but helps them to make decisions about their own well-being and health care choices [13,15]. Using the internet provides users with several resources that assist them in gaining information and insight into their conditions. Users of online resources will be able to engage in searching for information even after a consultation with a health care provider, as they may not have fully comprehended what was relayed during the session. Breast cancer patients who conducted personal research on the internet, in books, and via other media, for example, had improved knowledge of their conditions, and this proactive approach was deemed an independent predictor of active engagement in the choice of future therapy [16]. Unfortunately, low health literacy levels may influence how users, in general, are able to engage with information that is available online. This may lead to misunderstanding online information and, subsequently, inappropriate use of this information during health care decision making [17].

Health literacy has been defined as “the degree to which individuals have the capacity to obtain, process, and understand basic health information and the services needed to make appropriate health decisions” [18]. Poor health literacy has been linked to poorer health outcomes and increased health care costs and is, therefore, an important predictor of health status [19]. The quality of online health information in terms of readability and understandability is, therefore, an important consideration for both developers and consumers of these resources. Several evaluation tools are available to assess quality criteria such as the readability and understandability of health content that is available on the internet. Readability is “characterized according to the level of understanding a person must have to comprehend written materials, as determined by a set formula” [17] and may include algorithm systems, such as the Flesch-Kincaid Grade Level, the Simple Measure of Gobbledygook, or an overall electronic grading system that summarizes the various tools found online [20]. Another tool, the Patient Education Materials Assessment Tool, assesses the overall understandability and actionability of any audio-visual or written information for patients [21]. The Health On the Net code instrument [22] assesses the quality of information provided on websites [23], whereas web analytics services, like the Alexa analysis software, can be used to assess website visibility [24].

Previous studies evaluating online resources for stroke found that the quality, content, and readability of these websites were highly variable [1,2,13,24]. Even though internet connectivity and access to the internet through improved telecommunications, such as cellular phones and faster fiber-optic cables, have been improving, information on stroke will continue to remain inaccessible to users if it is not at an appropriate reading level [13]. Sharma et al [25] reported that “most consumer-oriented stroke information web pages were written at the 12th-grade level or above and that none complied with the...maximum recommended sixth-grade level.” In addition to readability, the accountability and reliability of education information on stroke websites have been investigated [2]. Accountability criteria entailed disclosure of authorship, ownership, and currency of information, while reliability was related to evidence-based practice as compared to the local clinical practice guidelines in the United Kingdom. Again, many stroke websites failed to conform to these quality standards, and few provided complete and accurate information regarding stroke [2]. Criteria such as trustworthiness (ie, timing, publisher, and contact information) and suitability for stroke prevention and self-management were explored more recently [1]. The Suitability Assessment of Materials instrument was used to evaluate content, literacy demands, graphics, layout, typography, learning stimulation and motivation, and cultural appropriateness for Korean Americans, including availability of multiple translations on the site. It is further recommended that the use of “graphical examples, multimedia, and interactive features can reduce the reading burden of stroke patients and caregivers, as well as build more confidence...when applying the information for condition management and rehabilitation in daily living” [1]. Examples include practical demonstration of behavior and daily skills, which the person with stroke could practice, cultivating greater self-efficacy, overall health, and well-being. Many different evaluation criteria and tools have been incorporated to assess online resources, with newer, more robust instruments continually being developed, to align with the evolving functionalities of this medium [26]. It has also been recommended that users and consumers should become partners in the co-design process of stroke-related websites to ensure that their needs are successfully met [2].

This study will, therefore, aim to map and evaluate freely available, current, online health information resources (ie, websites) that can empower people with stroke and their caregivers with information and self-help strategies poststroke. This comprehensive evaluation will review the content, readability, understandability, design, and quality characteristics of these online stroke health information resources.

## Methods

### Overview

When searching for educational information on stroke, information sources such as websites and social media pages are regularly accessed and will influence the knowledge and behavior of people with stroke and their caregivers. The search for online guidance has become a preferred strategy for many individuals globally, even more so during the COVID-19 pandemic, where fear, social distancing restrictions, and lack of accessible health services frequently influence their health-seeking behavior. Online resources reflect a new and current data source available to people with stroke and their caregivers in their search for information postincident. Following the rigorous approach of a scoping review framework will allow us to search, locate, and evaluate the contents and quality of these novel data sources in a systematic manner. The review will, therefore, be conducted according to a methodological framework for scoping reviews [27] involving five steps: (1) research question identification, (2) study identification, (3) study selection, (4) data charting, and (5) collating, summarizing, and reporting results.

### Review Framework

#### Overview

A scoping review approach will be used to locate, collate, and evaluate all relevant information on freely available online health information resources (ie, websites) that seek to empower people with stroke and their caregivers with information and self-help strategies poststroke. The reviewers will follow the guidelines of a scoping review methodological framework using a five-step process recommended by Levac et al [27], which is outlined below.

#### Step 1: Identifying the Review Question and Defining the Objectives

The initial stage of this review provides a roadmap for the entire process, as it clearly defines the breadth and depth of the scoping review process. The main constructs of the scoping review include synthesis of evidence relating to the information content, readability, understandability, and design characteristics of freely available online health information resources for stroke globally.

Therefore, the primary objectives of this scoping review will be to conduct the following:

1. Systematically search, summarize, and synthesize existing literature on the various freely available websites for stroke globally.
2. Describe these online resources in terms of typology (ie, type of resource) and geographic location.
3. Describe the information content in terms of its currency and credibility (ie, authoritativeness and trustworthiness).
4. Describe the design characteristics of these online health information resources in terms of readability, understandability, accessibility, aesthetics, navigability, interactivity, privacy, and data protection.
5. Identify exemplars of freely available websites for stroke globally. These exemplars may offer valuable insights and design elements to emulate for developers of new online resources for people with stroke and their caregivers.

#### Step 2: Searching, Eligibility Screening, and Selection of Relevant Online Stroke Resources

A structured online search will be conducted via Google by the primary researcher (GIJ) to obtain all freely accessible online educational resources and tools designed for people with stroke and their caregivers. Every step of the process will be recorded. In addition, websites of international organizations like the World Stroke Organization [28] will be specifically searched to identify potential online resources or links to other global stroke organizations or associations. The following combination of key search terms will be used: “stroke” AND “information,” “advice,” “help,” OR “support.”

The search will be conducted under the *private browsing* setting for the searches, in order to avoid being influenced by previous browsing history. The search will be limited to a time span of 2 years (2019 to 2021), representing the most recent and up-to-date online resources currently available to public users interested in this information (ie, people with stroke or those caring for survivors of stroke). Only the first three pages of results, containing 50 records each, generated by the search engine will be reviewed. This imitates the behavior of general internet users, where the majority (71.33%) may only view the first page, followed by fewer users (5.59%) looking at the second and third pages of search results [29]. Websites will be screened to identify resources that (1) contain information designed for people with stroke and their caregivers, (2) are available in the English language, and (3) do not have access or subscription charges.

Exclusion criteria include duplicate webpages, commercial sites or advertisements, commentary type webpages, and webpages that do not contain any relevant information about stroke or its management. Peer-reviewed primary literature will be excluded because it would likely exceed the comprehension and readability level of most patients and the general public. It is also assumed that most patients may not have access to scientific literature. Specific content for medical professionals will be excluded because of its intended target audience.

#### Step 3: Selection of Online Stroke Resources

One reviewer (GIJ) will screen the results generated via the Google search and apply the selection criteria to identify relevant websites. When in doubt, a final determination will be made through discussion with a second reviewer (TC) until consensus has been reached.

#### Step 4: Data Charting

Data will be extracted and captured on a custom Microsoft Excel spreadsheet. Extracted data items may include descriptions, content-related categories, and design-related categories, including but not restricted to typology, geographic location, credibility, and understandability. Definitions and descriptions of content and design characteristics are provided in Table 1 [26]. Data extracted will be cross-checked for completeness and accuracy.

**Table 1:** Definitions of quality criteria of online resources.

Criterion	Definition
Accessibility	Refers to “whether or not a site can be easily accessed. Widely used indicators included whether: a site is available, the links are active, special software is required for viewing the content, website contact information is clearly presented, and the site attends to users with disabilities” [26].
Aesthetics	Refers to “the look and feel of a site...major indicators are site layout (e.g., whether the layout is easy-to-follow, attractive, clear, simple, clean, and appealing), the use of images (e.g., whether they are relevant, appropriate, useful, and of high quality), and the use of headings (whether headings and subheadings are used)” [26].
Currency	Refers to “whether or not the content is up-to-date,” usually identified by the publication date and the time of the last update [26].
Credibility	Refers to “two components, authoritativeness and trustworthiness” [26].
Interactivity	Refers to the “capacity of a site to allow users to communicate with the system or with other users...including whether the site offers internal search functions, supports user input (e.g., commenting on content) and information exchange (e.g., chat rooms and links to social media), provides multimedia content, and personalizes content based on consumer characteristics” [26].
Navigability	Refers to “how easily a consumer can move around within a site...and includes whether the information architecture of a site is logical, supports easy navigation, and provides a site map” [26].
Privacy and data protection	Refers to “whether a site respects the privacy and confidentiality of personal data submitted by visitors.” Most studies used the indicator outlined in HONcode’s (Health On the Net Foundation Code of Conduct) privacy criterion; that is, the presence of policy statements describing what information is collected and how it is used—for example, whether users were given the opportunity to opt out of sharing personal information [26].
Readability	Refers to “whether or not the content of a site is understandable for general consumers without medical background” [26].
Cultural contextualization	For this review, the investigators will identify whether any of the online resources provide evidence of and/or information on aspects that were designed or adapted to make it culturally appropriate for diverse users. Some of the cultural indicators may include surface elements (eg, formats, pictures, and language) [26]. In addition, latent messages and themes (eg, whether examples for patients from diverse sociodemographic backgrounds are included) will be identified.

As the field of digital health is expanding, so is the plethora of evaluation tools and criteria checklists available to assess the quality of online resources and webpages. The researcher is interested in whether the information content included in these online stroke information resources is comprehensive, current, and evidence based, as well as whether appropriate and accessible formats are being used. The description of the contents of each data source may include currency and credibility of the information, while design characteristics may include readability, understandability, accessibility, aesthetics, navigability, interactivity, privacy, and data protection. Data items will be extracted and summarized narratively and, where available, appraisal tools will be used. Selection of the appraisal tool will be determined by the specific criteria or characteristics to be appraised. Table 2 provides more details of the various quality criteria and indicators, along with appraisal tools or systems as described by Zhang et al [26].

**Table 2:** Quality criteria and indicators to assess online information sources and resources, as described by Zhang et al [26].

Characteristics and criteria			Examples of indicators		Validated evaluation tools (where applicable)
**Content-related characteristics**					
	Currency	Publication date Time of last update		N/A^a^	
	Credibility (authoritativeness and trustworthiness)	Authorship Author name and professional credentials Editorial process Site domain and site type Disclosure Aims of the website Owner or sponsor of the site Financial disclosure and conflict of interest Contact information disclosed Advertising policy Target audience disclosed Bias disclosed Attribution Source of the content and references Additional source of support Copyright, logo, or page title disclosed Links to other related sites Third-party accreditation Health On the Net certified Site popularity Page rank in search engine results list Site traffic statistics Presentation of the content Balanced content Spelling errors		HONcode (Health On the Net Foundation Code of Conduct) conformity developed by the Health On the Net Foundation Alexa web analytics software to assess domain popularity and visibility	
**Design-related characteristics**					
	Readability	Site content should be understandable for general consumers without medical background		Patient Education Materials Assessment Tool	
	Accessibility	Operational: sites available, no dead links, and browser independent Clear presentation of website contact information Registration and accessing fee Accessible for people with disabilities (eg, font size and graphics with captions) Other languages offered Website technical support available		N/A	
	Aesthetics	Site layout Appropriate use of images Use of headings Color schema Design consistency		N/A	
	Navigability	Navigation structure: information presented in a logical order and easy navigation between links Site map		N/A	
	Interactivity	Information exchange (eg, forums and emails) Internal search engines Multimedia capability FAQ (frequently asked questions) section Personalization		N/A	
	Privacy and data protection	Policy on the collection and use of personal data		N/A	

^a^N/A: not applicable; there were no validated evaluation tools for this criterion.

#### Step 5: Collating, Summarizing, and Reporting Results

One reviewer (GIJ) will extract all the information related to content and design characteristics of stroke websites and will cross-check the entries in a reviewer-developed Microsoft Excel data sheet. A random selection of 10% of the extracted data will be checked by a second reviewer (TC). After comparison, any discrepancies will be resolved via discussion between the reviewers; if necessary, a third reviewer (KG) will be consulted. The extracted data will be summarized narratively using text and tables; where appropriate, thematic content analysis will be employed.

## Results

As of February 2021, the scoping review is in the data extraction stage. Data will be synthesized, and the first results are expected to be submitted for publication in an open-access peer-reviewed journal in August 2021. We will develop an accessible summary of the results for stakeholder meetings. Ethical approval is not required for this scoping review, as it will only include publicly available information or data. Data generated from this review will be made available upon reasonable request.

## Discussion

### Potential Limitations of the Review

Only English-language websites will be reviewed in this study. This may result in missing potentially good-quality resources available in other languages as well as excluding end users with limited English proficiency. Even though Google is one of the most common and well-known search engines available internationally, limiting the search to only this search engine may be a limitation of our study. The use of computer-based analysis of readability may overestimate the difficult level of online information on websites, but more than one readability index will be used in this review to provide a broader interpretation of this quality criterion.

### Conclusions

This review will attempt to map and evaluate the quality of the content, readability, and design of current, freely available, online health information resources (ie, websites) that empower people with stroke and their caregivers with information and self-help strategies poststroke, globally. Comprehensively mapping existing resources will assist developers with gaining insight into gaps across a range of quality criteria. The results of this review will be used to identify exemplar online health information resources of good quality and which specific aspects may need to be improved. An evaluation of these exemplar online stroke resources by target users will be incorporated in a follow-up study to validate the obtained results of the review from the user’s perspective. The exemplar online stroke resources, identified during the review, will also inform the design of a South African–specific stroke-related digital health intervention. The envisaged co-design of the new contextually appropriate digital health intervention will, therefore, be informed by explicit quality criteria, international exemplar information resources, and, finally, input from end users, including people with stroke, their caregivers, and health care professionals involved in stroke care in South Africa.

## Abbreviations

DALY: disability-adjusted life year
NRF: National Research Foundation
